# Travis County Medical Society. North West Texas Medical Association, Etc.

**Published:** 1890-09

**Authors:** 


AT A MEETING of the Travis County Medical Society, held at Austin, September 4th, inst., the following officers were elected for the ensuing year:

President, Dr. B. F. Church; Vice President, Dr. F. Litten; Secretary, Dr. J. Cummings.

---

The Northwest Texas Medical Association, which was organized in Abilene in June last, will hold its regular quarterly meeting at Cisco, 7th of October.

The following papers will be read and discussed: "Management of Placenta in Natural Labor."—Dr. Wallace. "Continued Fever."—Dr. Keown. "Drainage of Wounds." —Dr. Hollis.

**What can we do with Lingering Labors?**—[A letter from Dr. Geo. C. Masker, at Birmingham, to The Medical Record.] In the Section of Obstetrics and Gynecology in the British Medical Association, a most interesting discussion upon the subject of "Management of Lingering Labor," a paper read by Dr. W. S. Playfair, brought out a striking variety of opinions concerning the use of drugs in this class of cases, which, Dr. Playfair was careful to explain in beginning, included only cases not attributable to mechanical obstruction, but simple due to uterine inertia. Dr. Playfair said that in his opinion, based on his own experience and corroborated by the views entertained by the authorities of the leading maternity hospitals of Great Britain, the use of ergot prior to the expulsion of the placenta, was practically obsolete. He relies more upon position, pressure over abdomen, and considers chloral hydrate of drugs the sheet anchor; to be used up to the time the head presses upon the perinæum, then he uses chloroform.

Dr. T. More Madden said, his practice was to use ergot in doses of three or four drachms in the beginning of the second stage of labor, and usually to supplement this with a hypodermic injection of ergotin.

Dr. Cameron carries two grain pills of opium in his pocket, and if any delay occurs gives a pill, which he expects will put the patient to sleep for a number of hours or speedily terminate the case. Certainly a remarkable variance among authorities.

